# Heat Shock-Induced Dephosphorylation of Eukaryotic Elongation Factor 1BδL by Protein Phosphatase 1

**DOI:** 10.3389/fmolb.2020.598578

**Published:** 2021-01-14

**Authors:** Taku Kaitsuka, Kazuhito Tomizawa, Masayuki Matsushita

**Affiliations:** ^1^Department of Molecular Physiology, Faculty of Life Sciences, Kumamoto University, Kumamoto, Japan; ^2^School of Pharmacy in Fukuoka, International University of Health and Welfare, Okawa, Japan; ^3^Department of Molecular and Cellular Physiology, Graduate School of Medicine, University of the Ryukyus, Okinawa, Japan

**Keywords:** translation elongation factor, stress response, protein phosphorylation, phosphatase, tissue-specific transcription factor, heat-shock

## Abstract

Several variant proteins are produced from *EEF1D*, including two representative proteins produced via alternative splicing machinery. One protein is the canonical translation eukaryotic elongation factor eEF1Bδ1, and the other is the heat shock-responsive transcription factor eEF1BδL. eEF1Bδ1 is phosphorylated by cyclin-dependent kinase 1 (CDK1), but the machinery controlling eEF1BδL phosphorylation and dephosphorylation has not been clarified. In this study, we found that both proteins were dephosphorylated under heat shock and proteotoxic stress, and this dephosphorylation was inhibited by okadaic acid. Using proteins with mutations at putative phosphorylated residues, we revealed that eEF1Bδ1 and eEF1BδL are phosphorylated at S133 and S499, respectively, and these residues are both CDK1 phosphorylation sites. The eEF1BδL S499A mutant more strongly activated *HSPA6* promoter-driven reporter than the wild-type protein and S499D mutant. Furthermore, protein phosphatase 1 (PP1) was co-immunoprecipitated with eEF1Bδ1 and eEF1BδL, and PP1 dephosphorylated both proteins *in vitro*. Thus, this study clarified the role of phosphorylation/dephosphorylation in the functional regulation of eEF1BδL during heat shock.

## Introduction

Eukaryotic translation elongation factor 1BδL (eEF1BδL) is an alternative splicing variant of eEF1Bδ. Canonical eEF1Bδ (referred to as eEF1Bδ1) functions as a guanine nucleotide exchange factor for eEF1A (Le Sourd et al., 2006), whereas eEF1BδL functions as a heat shock and proteotoxic stress-responsive transcription factor for heat-shock element (HSE)-containing genes (Kaitsuka et al., 2011). eEF1BδL is a mammalian and avian-specific splicing variant, and its expression is restricted to the brain and testis in mice (Kaitsuka et al., 2011; Kaitsuka and Matsushita, 2015). Concerning its physiological roles in humans, *EEF1D* encodes several proteins, and mutations in this gene have been found in patients with severe intellectual disability and revealed to be related to neurodevelopmental disorders (Reuter et al., 2017; Ugur Iseri et al., 2019). Furthermore, we previously reported that deletion of the exon specific to eEF1BδL resulted in audiogenic seizures in mice (Kaitsuka et al., 2018), suggesting that this protein is important for intact brain function. As mentioned previously, the relationship of this protein with human disease and the phenotype of eEF1BδL-knockout mice have gradually been revealed, but the molecular mechanisms by which eEF1BδL is activated and recruited to HSE-containing genes during stress are poorly understood.

Protein phosphorylation and dephosphorylation are fundamental principles guiding cellular function (Leslie and Nairn, 2019). Canonical eEF1Bδ1 is phosphorylated at S133 by cyclin-dependent kinase 1 (CDK1)/cyclin B kinase, and its phosphorylation is proposed to result in an inhibitory effect on translation elongation, leading to the inhibition of protein synthesis (Monnier et al., 2001; Le Sourd et al., 2006). However, a phosphatase for this protein has not been identified. Additionally, it is unclear whether eEF1BδL is similarly phosphorylated by such kinases. In this study, we attempted to identify the kinase and phosphatase of eEF1BδL and clarify the machinery of eEF1BδL activation during heat shock. We found that both eEF1Bδ1 and eEF1BδL were dephosphorylated under heat shock and proteotoxic stress at S133 and S499, respectively, and the phosphorylation-deficient mutant of eEF1BδL exhibited higher transcriptional activity for HSE-containing genes. Furthermore, protein phosphatase 1 (PP1) dephosphorylated these proteins at the aforementioned serine residues *in vitro*. Our study provides new insights into the regulation of eEF1BδL activity under stresses by phosphorylation and dephosphorylation.

## Materials and Methods

### Cell Culture and Treatment

Primary cultured neurons were obtained from the hippocampi of fetal C57BL/6J mice (CLEA Japan, Inc., Tokyo, Japan) on embryonic day 17 and maintained in neurobasal medium containing 2% B-27 supplement (Thermo Fisher Scientific, Waltham, MA, USA) at 37°C in a 5% CO_2_ atmosphere. For heat shock exposure, neurons were incubated in a 42°C water bath. MG132 (FUJIFILM Wako Pure Chemical Corporation, Osaka, Japan) was applied to neurons at a concentration of 10 μM. OA and CysA were added 2 h before heat shock at concentrations of 100 nM and 5 μM, respectively. HEK293 cells were maintained in Dulbecco's modified Eagle's medium (Thermo Fisher Scientific) containing 10% fetal bovine serum (Thermo Fisher Scientific) at 37°C in an atmosphere of 5% CO_2_.

### Transfection, Luciferase-Based Reporter Assay, and Quantitative PCR Analysis

The mammalian expression plasmids Flag-eEF1Bδ1 and Flag-eEF1BδL were cloned as described previously (Kaitsuka et al., 2011). The mutants of their phosphorylation sites were prepared by site-directed mutagenesis using Pfu DNA polymerase (Agilent, Santa Clara, CA, USA). The *HSPA6* promoter-luciferase plasmid was cloned as described previously (Kaitsuka et al., 2011), and a plasmid carrying the *Renilla* luciferase gene driven by the thymidine kinase promoter was obtained from Promega (Madison, WI, USA). Transfections were performed using Lipofectamine LTX reagent (Thermo Fisher Scientific) according to the manufacturer's instructions. Twenty-four hours after transfection, cells were lysed, and luminescence was determined using dual-luciferase assay reagent (Promega). For quantitative PCR analysis, total RNA was extracted using TRIzol reagent (Thermo Fisher Scientific, Waltham, MA, USA). cDNA was prepared by reverse transcription of 500 ng of total RNA using the PrimeScript™ RT reagent Kit (Takara Bio, Shiga, Japan). The resulting cDNAs were amplified using FastStart Essential DNA Green Master (Roche Applied Science, Indianapolis, IN, USA) and analyzed with a LightCycler® 96 Real-Time PCR System (Roche Applied Science). mRNA expression data were normalized to the *ACTB* expression in a corresponding sample. The primers used are as follows: human *HSPA6* (forward, TCCAGCATCCGACAAGAAGCT; reverse, TGCTTCATGTCCGACTGCACC) and human *ACTB* (forward, CCTCATGAAGATCCTCACCGA; reverse, TTGCCAATGGTGATGACCTGG).

### Western Blot Analysis and Immunoprecipitation

Cells were washed in phosphate-buffered saline (PBS), lysed in SDS sample buffer (50 mM Tris-HCl, pH 6.8, 2% SDS, 6% β-mercaptoethanol, 10% glycerol, 0.005% bromophenol blue), and boiled for 5 min. Samples were subjected to SDS-PAGE and then transferred to polyvinylidene difluoride membranes. Membranes were pretreated with Blocking One (Nacalai Tesque, Kyoto, Japan) and then probed with the following antibodies: rabbit anti-EEF1D (eEF1Bδ) (1:5,000; 10630-1-AP, Proteintech Group, Rosemont, IL, USA), mouse anti-β-actin (1:10,000; M177-3, Medical and Biological Laboratories, Nagoya, Japan), mouse anti-Flag M2 (1:10,000; F1804, Sigma-Aldrich, St. Louis, MO, USA), mouse anti-GFP (1:2,000; M048-3, Medical and Biological Laboratories), mouse anti-glutathione-*S*-transferase (GST) (1:5,000; 013-21851, FUJIFILM Wako Pure Chemical Corporation), rabbit anti-PP1 (1:1,000; Merck Millipore, Burlington, MA, USA), rabbit anti-PP2A (1:1,000; 07-324, Merck Millipore), rabbit anti-EEF1B2 (eEF1Bα) (1:1,000; 10483-1-AP, Proteintech Group), rabbit anti-eEF2 (1:1,000; 2332S, Cell Signaling Technology, Danvers, MA, USA), mouse anti-Hdac2 (1:1,000; 5113S, Cell Signaling Technology), rabbit anti-GAPDH (1:10,000; GTX100118, GeneTex, Irvine, CA, USA), rabbit anti-Nrf2 (1:200; SC-13032, Santa Cruz Biotechnology, Dallas, TX, USA), and rabbit anti-phospho-eEF1BδL/1 (S499/S133) (1:1,000; produced by Sigma Genosys; Sigma-Aldrich). Then, the membranes were washed and incubated with a horseradish peroxidase-conjugated anti-rabbit IgG antibody (Thermo Fisher Scientific) or anti-mouse IgG antibody (Dako, Carpinteria, CA, USA). The membranes were washed and incubated with Amersham ECL prime Western blotting detection reagent (GE Healthcare, Piscataway, NJ, USA), and the blots were visualized using an ImageQuant400 system (GE Healthcare).

For immunoprecipitation, cells were washed with PBS and lysed in NP lysis buffer (50 mM Tris-HCl, pH 7.5, 120 mM NaCl, 0.5% NP-40, 1 mM EDTA, 50 μg/ml aprotinin, 50 μg/ml leupeptin, 1 mM PMSF) at 4°C for 30 min, and then the insoluble materials were removed via centrifugation at 20,000 × g for 10 min. Cell extracts were incubated with anti-EEF1D or anti-GFP antibody overnight at 4°C, followed by incubation with Protein A or Protein G Sepharose Fast Flow (GE Healthcare). After 1 h of incubation, the beads were washed three times with NP buffer and once with Tris-buffered saline (50 mM Tris-HCl, pH 7.5, 120 mM NaCl). Proteins bound to the beads were eluted in SDS sample buffer, and the eluted proteins were subjected to Western blot analysis with the appropriate antibodies.

### Phosphorylation and Dephosphorylation Assay

Recombinant GST-tagged eEF1BδL and eEF1Bδ1 proteins were purified as described previously (Kaitsuka et al., 2011). Briefly, *Escherichia coli* BL21 (DE3; Thermo Fisher Scientific) transformed with each plasmid was grown at 37°C to reach an optical density at 600 nm of 0.8, and then isopropyl-β-d-thiogalactopyranoside (Sigma-Aldrich) was added to a final concentration of 0.2 mM. Then, the bacteria were cultured for 6 h at 32°C and lysed in lysis buffer (50 mM Tris-HCl, pH 8.0, 300 mM NaCl, 1 mM EDTA, 1 mg/ml lysozyme, 2 mM DTT, 0.8% NP40) containing protease inhibitor cocktail (Sigma-Aldrich). The supernatant was recovered via centrifugation, and GST-tagged proteins were purified using glutathione Sepharose (GE Healthcare). Finally, tagged GST was removed via cleavage using PreScission Protease (GE Healthcare).

The phosphorylation assay was performed using the CDK1/Cyclin A2 Kinase Enzyme System (Promega), and the phosphorylated proteins were analyzed by Western blot analysis using the appropriate antibodies. The dephosphorylation assay was performed using CIP (New England Biolabs, Ipswich, MA, USA) or recombinant PP1 catalytic subunit, α-isoform (Sigma-Aldrich) in dephosphorylation buffer (20 mM Tris-HCl, pH 7.5, 0.1 mM MnCl_2_, 1 mM DTT, 0.1 mg/ml bovine serum albumin), and then phosphorylated proteins were analyzed by Western blot analysis using the appropriate antibodies.

### Statistical Analysis

All results were confirmed by more than three independent experiments. The data in the graphs are expressed as the mean ± SEM. Comparisons between multiple groups were made using one-way analysis of variance followed by Dunnett's *post-hoc* analysis.

### An Animal Study

All procedures involving mice were performed in compliance with the National Institutes of Health and Kumamoto University Animal Facility guidelines, and protocols were approved by the Laboratory Animal Care and Use Committees of Kumamoto University.

## Results

### eEF1Bδ1 and eEF1BδL Are Dephosphorylated Under Heat Shock and Proteotoxic Stress

**Figure 1:**
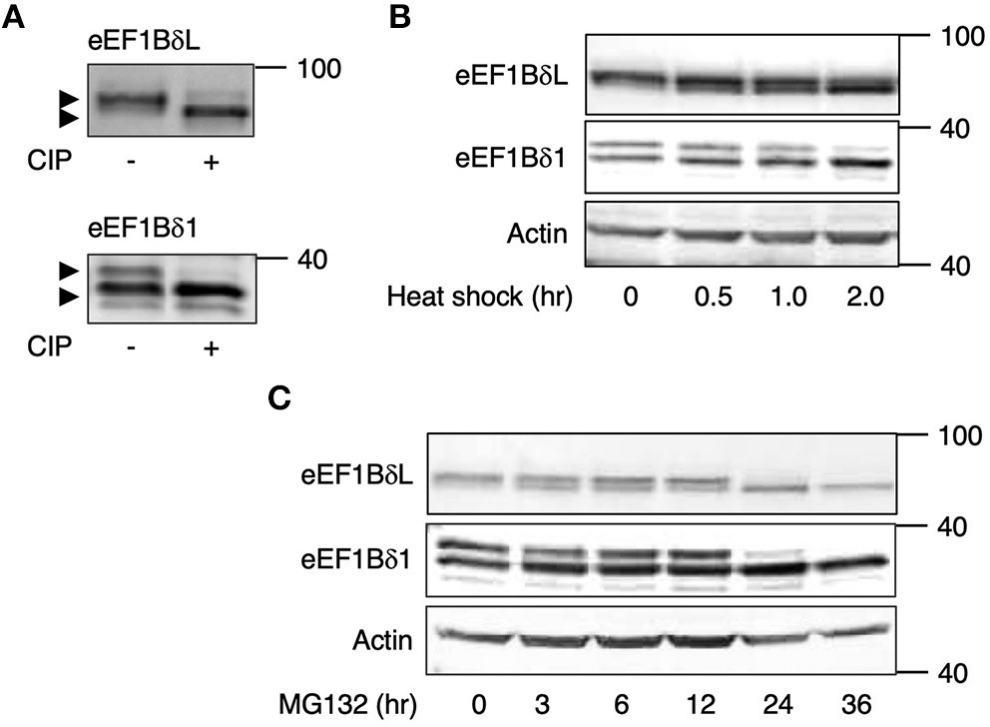
eEF1Bδ is dephosphorylated during stress exposure. **(A)** Immunoprecipitated eEF1Bδ proteins from cultured mouse neurons were treated with calf-intestinal alkaline phosphatase (CIP). The upper bands disappeared following CIP treatment, indicated that they represent the phosphorylated proteins. Similar results were obtained in three independent experiments. **(B,C)** Cultured mouse hippocampal neurons were exposed to heat shock (42°C) **(B)** or treated with 10 μM MG132 **(C)** for the indicated periods. The abundance of the upper bands of eEF1BδL and eEF1Bδ1 decreased following these treatments. Similar results were obtained in three independent experiments.

The total lysates from mouse cultured hippocampal neurons were subjected to SDS-PAGE followed by Western blot analysis using anti-eEF1Bδ antibody, and the eEF1Bδ1 and eEF1BδL bands were both accompanied by an upwardly shifted band. The shifted band of eEF1Bδ1 has been identified as a phosphorylated form (S133) (Mulner-Lorillon et al., 1994; Sivan et al., 2011). Therefore, we tested whether the shifted band of eEF1BδL is also a phosphorylated form using immunoprecipitated proteins from mouse cultured neurons and calf-intestinal alkaline phosphatase (CIP). As expected, the shifted band of eEF1BδL was abolished by CIP treatment, illustrating that this shift was caused by phosphorylation (Figure 1A). A similar result was obtained when eEF1Bδ1 was subjected to the same experiment (Figure 1A).

To examine whether the phosphorylation state of eEF1BδL and eEF1Bδ1 is affected by heat shock, we exposed mouse cultured hippocampal neurons to heat (42°C). During this treatment, the phosphorylated forms of eEF1BδL and eEF1Bδ1 gradually decreased in abundance, with remarkable reductions observed after 2 h (Figure 1B). The proteasome inhibitor MG132 elicits proteotoxic stress by increasing misfolded protein levels by inhibiting protein degradation (Bush et al., 1997). Treatment with MG132 also decreased the levels of their phosphorylated forms within 24 h (Figure 1C).

**Figure 2:**
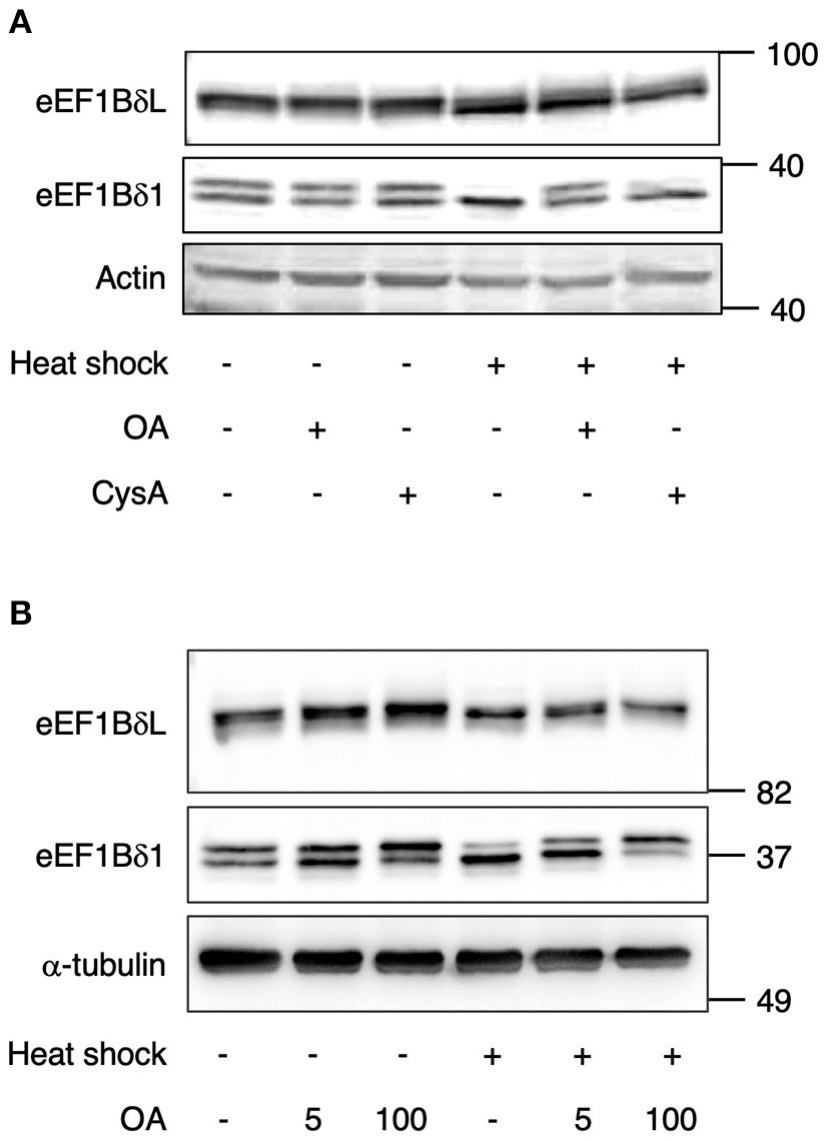
Dephosphorylation of eEF1Bδ is inhibited by okadaic acid (OA). **(A)** Cultured mouse hippocampal neurons were pretreated with 100 nM okadaic acid (OA) or 5 μM cyclosporine A (CysA) for 2 h followed by heat shock for 2 h. The abundance of the upper bands of eEF1BδL and eEF1Bδ1 decreased following heat shock in DMSO- and CysA-treated neurons but not in OA-treated neurons. Similar results were obtained in three independent experiments. **(B)** Cultured mouse hippocampal neurons were pretreated with 5 and 100 nM OA for 2 h followed by heat shock for 2 h. The decrease of the upper bands of eEF1BδL and eEF1Bδ1 following heat shock was not fully repressed in 5 nM OA-treatment. Similar results were obtained in three independent experiments.

We assumed that some PP might dephosphorylate these proteins under heat stress. To examine this assumption, the neurons were pretreated with the phosphatase inhibitors okadaic acid (OA; PP1 and PP2A inhibitor) and cyclosporine A (CysA; PP2B inhibitor) and then exposed to heat shock. Heat shock-induced downregulation of their phosphorylated forms was inhibited by OA pretreatment, but not by CysA pretreatment (Figure 2A), suggesting that PP1 or PP2A dephosphorylates eEF1Bδ1 and eEF1BδL under such stress. Because the sensitivity to OA of PP2A is higher than PP1, the effect of lower concentration (5 nM) of OA was tested. 5 nM OA pretreatment partially inhibited the heat shock-induced dephosphorylation of eEF1BδL and eEF1Bδ1 (Figure 2B), illustrating that PP1 is predominant phosphatase of both proteins.

### S499 of eEF1BδL (S133 of eEF1Bδ) Is Dephosphorylated Under Heat Shock

**Figure 3:**
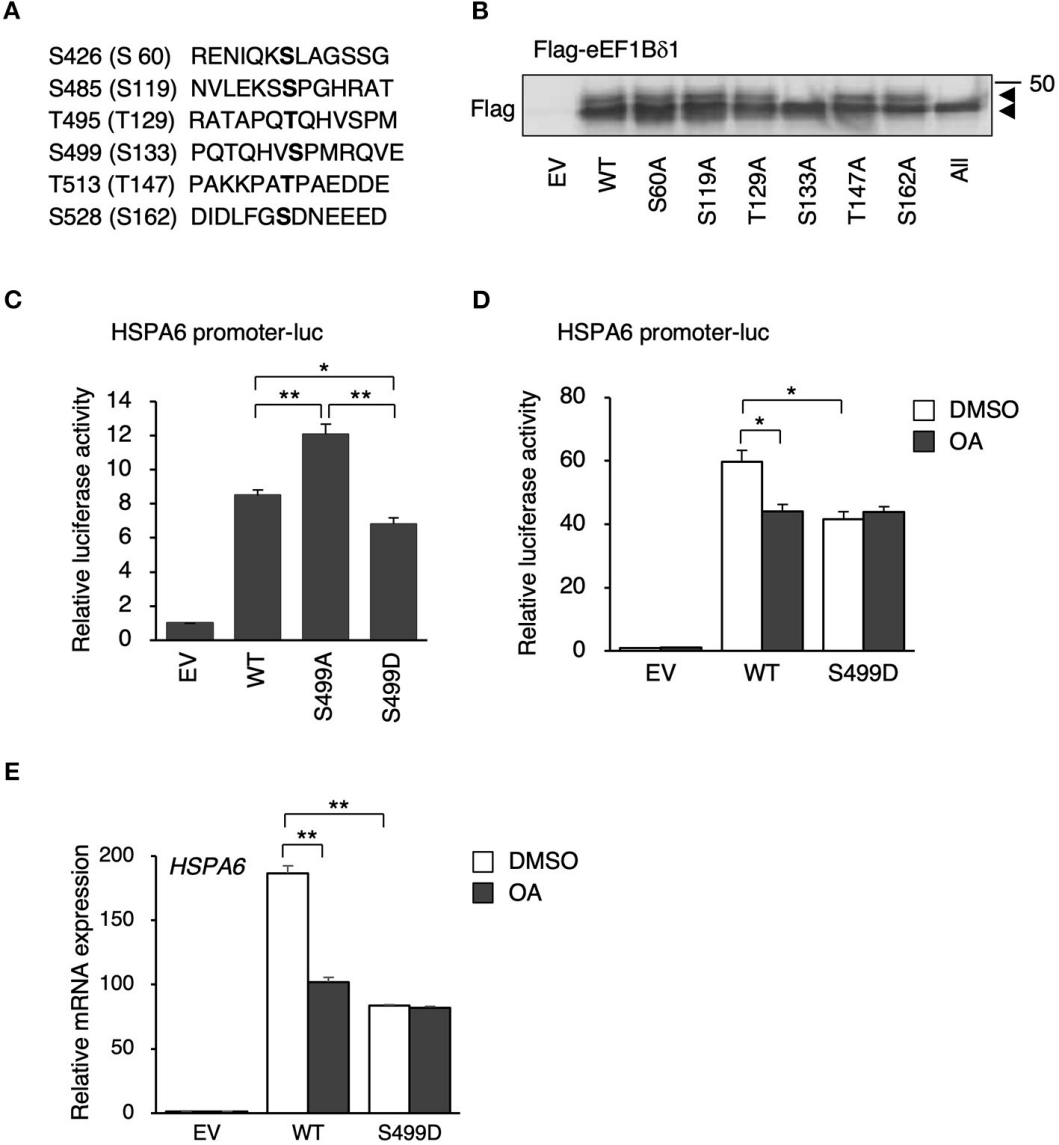
S133 of eEF1Bδ1 and S499 of eEF1BδL are dephosphorylated following heat shock. **(A)** Amino acid sequences of candidate phosphorylation sites in eEF1BδL. The residues in parentheses correspond to those in eEF1Bδ1. **(B)** HEK293 cells were transfected with the expression plasmids carrying phospho-mutants of eEF1Bδ1 protein and then subjected to Western blotting. “All” means the mutants carrying all putative phosphorylation site mutated to alanine, An upper band was not detected for eEF1Bδ1 S133A. Similar results were obtained in three independent experiments. EV, empty vector. **(C)** HEK293 cells were transfected with the expression plasmids carrying green fluorescent protein (GFP)-tagged S499 phospho-mutants of eEF1BδL and *HSPA6* promoter-driven luciferase and then subjected to a luciferase reporter assay. The reporter activity in eEF1BδL S499A-expressing cells was higher than that in wild-type (WT)- and S499D-expressing cells. *p < 0.05, **p < 0.01, *n* = 3 for each, Student's *t*-test. **(D)** HEK293 cells were transfected with the expression plasmids carrying Flag-tagged eEF1BδL WT, S499D mutant, and *HSPA6* promoter-driven luciferase and then treated with 20 nM okadaic acid (OA) for 12 h and subjected to a luciferase reporter assay. The reporter activity in eEF1BδL WT-expressing cells was repressed by OA treatment, while that activity in S499D-expressing cells was not repressed by this treatment. *p < 0.05, *n* = 3 for each, Student's *t*-test. **(E)** HEK293 cells were transfected with the expression plasmids carrying Flag-tagged eEF1BδL WT and S499D mutant, then treated with 20 nM okadaic acid (OA) for 12 h and subjected to a quantitative PCR assay. The *HSPA6* expression significantly induced in eEF1BδL WT-expressing cells, and this induction was repressed by OA treatment, while that induction in S499D-expressing cells was not repressed by OA treatment. **p < 0.01, *n* = 6 for each, Student's *t*-test.

eEF1Bδ1 has defined (S133; Mulner-Lorillon et al., 1994) and undefined putative phosphorylation sites (S60, S119, T129, T147, and S162) as listed in the UniProtKB database (P29692 version 94) (Figure 3A). These residues correspond to S426, S485, T495, S499, T513, and S528 of eEF1BδL, respectively. We prepared expression plasmids carrying mutants in which each serine or threonine was replaced by alanine to mimic the dephosphorylated states of eEF1Bδ1 and eEF1BδL. Western blot analysis of the lysate of HEK293 cells expressing each eEF1Bδ1 mutant revealed that the S133A mutant and mutant carrying all phosphorylation sites mutated did not feature the upwardly shifted band, illustrating that S133 (corresponding to S499 of eEF1BδL) is dephosphorylated under heat shock (Figure 3B). Next, we assessed the transcriptional activity of eEF1BδL wild-type (WT), S499A, and S499D (a phosphorylated mutant) using plasmids carrying *HSPA6* promoter-driven luciferase. The mutation to negatively charged aspartic acid can mimic the phosphorylated state, because phosphorylation generally adds negative charge to the target residue (Pearlman et al., 2011). Luciferase activity was significantly higher in the S499A mutant-expressing cells than in the S499D- and WT-expressing cells (Figure 3C). Additionally, this activity of S499D-expressing cells was significantly lower than of WT-expressing cells, illustrating that eEF1BδL phosphorylation at S499 reduces its transcriptional activity. To test the effect of OA treatment on *HSPA6* transcriptional activity of eEF1BδL, eEF1BδL WT-expressing cells were treated with OA for 12 h and the luciferase assay was performed (Figure 3D). As a result, this activity induced by eEF1BδL WT was significantly repressed by OA, while this activity was not affected in S499D mutant-expressing cells by OA, illustrating that OA affect the transcriptional activity of eEF1BδL via S499 residue. Similar result was confirmed by *HSPA6* mRNA expression in eEF1BδL WT- and S499D mutant-expressing cells (Figure 3E).

### S499 of eEF1BδL Is Phosphorylated by CDK1

**Figure 4:**
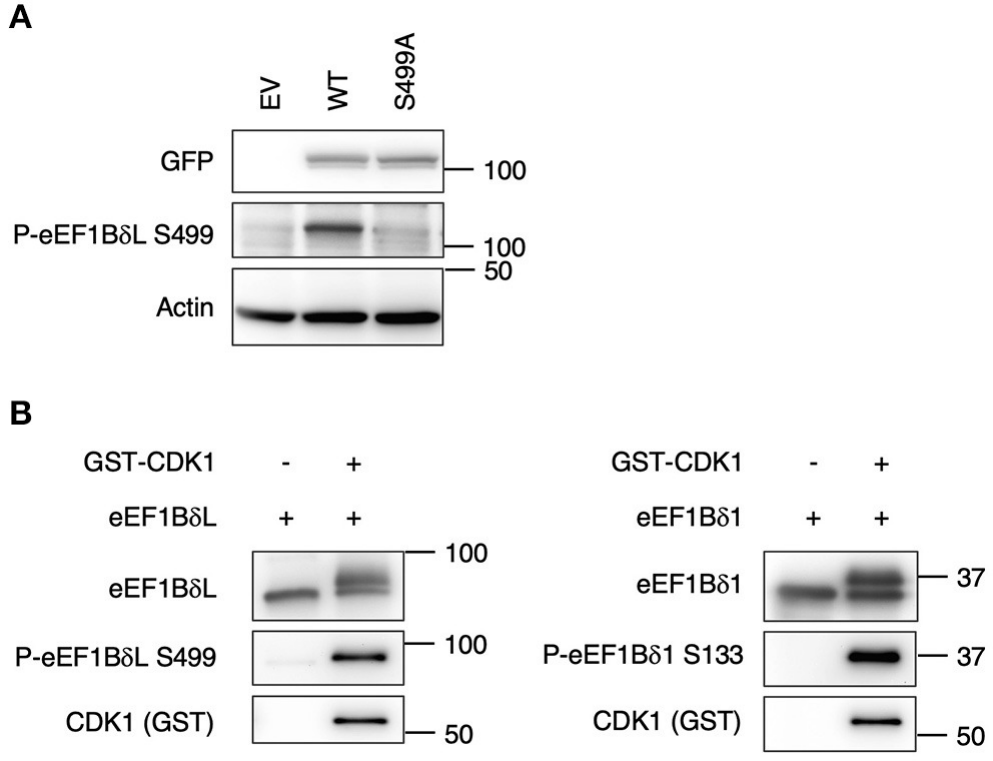
S133 of eEF1Bδ1 and S499 of eEF1BδL are cyclin-dependent kinase 1 (CDK1) phosphorylation sites. **(A)** Validation of the antibody against phosphorylated eEF1BδL (S499). HEK293 cells were transfected with the expression plasmid carrying the S499A phospho-mutant of eEF1BδL tagged with GFP. Then cells were lysed and subjected to Western blotting. GFP, green fluorescent protein. Similar results were obtained in three independent experiments. **(B)** Recombinant eEF1Bδ1 and eEF1BδL were reacted with recombinant glutathione S-transferase-tagged CDK1. Upwardly shifted bands appeared in the presence of CDK1. The phospho-eEF1Bδ-specific antibody clearly detected the phosphorylated forms of eEF1BδL (left) and eEF1Bδ1 (right). Similar results were obtained in three independent experiments. GST, glutathione-*S*-transferase.

To detect eEF1BδL dephosphorylation at S499 specifically, we raised an antibody against a synthetic phosphorylated peptide (PQTQHVpSPMRQVE). To confirm its specificity, Western blot analysis was performed on the lysate of HEK293 cells expressing eEF1BδL WT and S499A, revealing that the band corresponding to the WT protein was clearly abolished in the S499A lysate (Figure 4A). In addition, it was confirmed that this antibody reacted with the upwardly shifted band of eEF1Bδ1 which disappeared by CIP treatment *in vitro* and by heat shock *in vivo* (Supplementary Figures 1A,B). Next, we examined whether S499 is phosphorylated by CDK1 via an *in vitro* phosphorylation assay because S133 of eEF1Bδ1 is phosphorylated by this kinase. When the reaction mixture was subjected to Western blotting using anti-phosphorylated S499 eEF1BδL antibody, a band dramatically appeared in the presence of CDK1. This result was accompanied by the appearance of an upwardly shifted band when reacted with anti-total eEF1Bδ antibody (Figure 4B). Similar results were obtained when eEF1Bδ1 protein was subjected to this CDK1 phosphorylation assay, consistent with previous reports (Monnier et al., 2001).

### PP1 Is Co-immunoprecipitated With eEF1BδL and eEF1Bδ1 and Dephosphorylates Them *in vitro*

**Figure 5:**
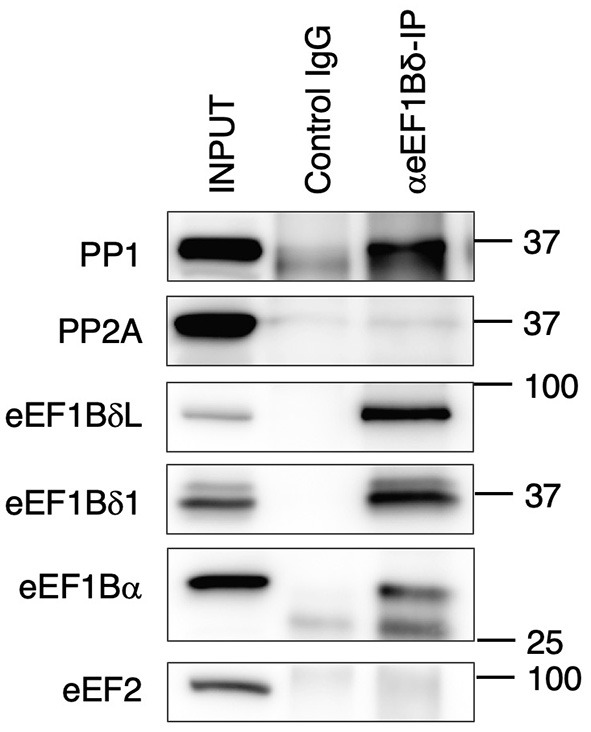
Protein phosphatase 1 (PP1) exists in eEF1Bδ co-immunoprecipitates. Neuro-2a cells were lysed and then immunoprecipitated with anti-eEF1Bδ antibody. Both eEF1BδL and eEF1Bδ1 were precipitated by this antibody, and PP1, but not PP2A, was detected in the immunoprecipitate. eEF1Bα and eEF2 were used as the positive and negative controls, respectively. Similar results were obtained in three independent experiments.

As mentioned previously, OA is a potent inhibitor of PP1 and PP2A. Therefore, we tested whether eEF1BδL and eEF1Bδ1 interact with those phosphatases via a co-immunoprecipitation assay followed by Western blotting. When eEF1Bδ proteins and their interactors were immunoprecipitated with anti-eEF1Bδ antibody, PP1, but not PP2A, was detected in the immunoprecipitates (Figure 5).

**Figure 6:**
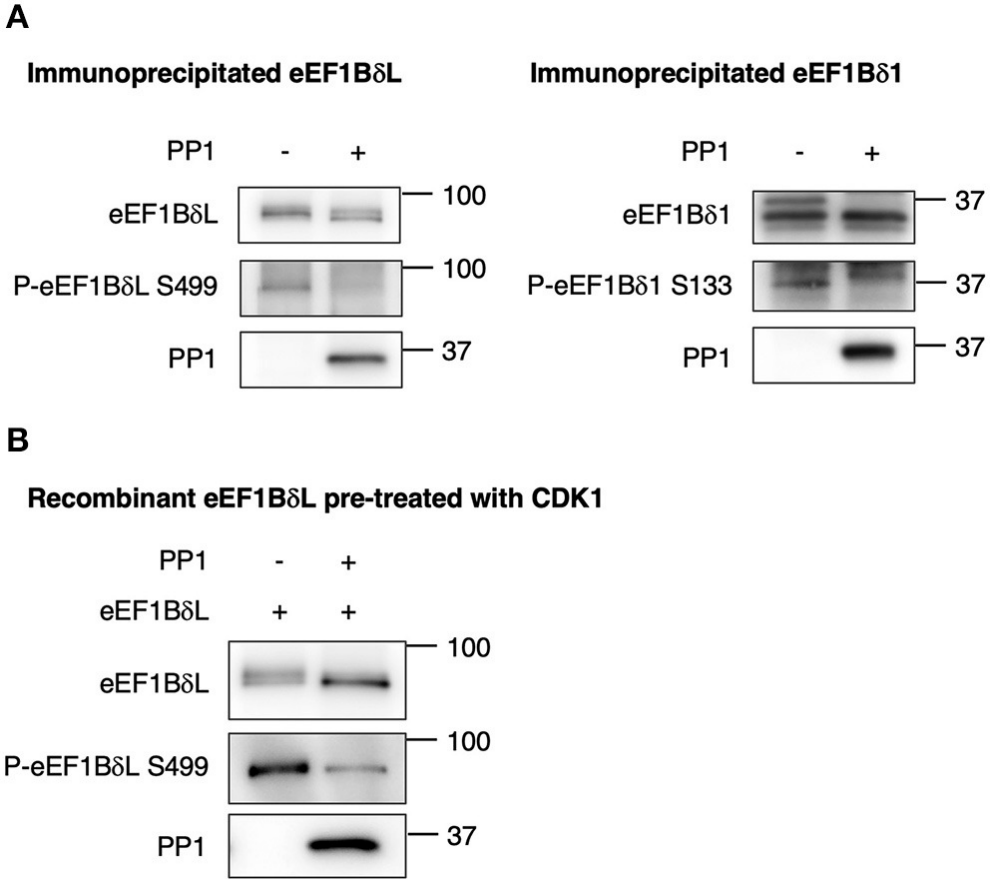
Protein phosphatase 1 (PP1) dephosphorylates eEF1Bδ. **(A)** eEF1BδL and eEF1Bδ1 proteins were purified via immunoprecipitation from mouse brain tissue using an eEF1Bδ antibody in a stringent condition, and the dephosphorylation reaction was performed with or without recombinant PP1. Upwardly shifted bands disappeared in the presence of PP1 for both eEF1BδL (left) and eEF1Bδ1 (right). The bands detected by the phospho-specific antibody also disappeared. Similar results were obtained in three independent experiments. **(B)** Recombinant eEF1BδL was reacted with glutathione-*S*-transferase-tagged cyclin-dependent kinase 1 (GST-CDK1) to assess its phosphorylation. Then, GST-CDK1 was removed from the reaction mixture, and the remaining eEF1BδL protein was treated with or without PP1. Upwardly shifted bands decreased in the presence of PP1. Similar results were obtained in three independent experiments.

Next, to prove the direct dephosphorylation by PP1, eEF1BδL, and eEF1Bδ1 were purified via immunoprecipitation from mouse brain tissue and reacted with recombinant PP1 protein. Both upper shifted blots (phosphorylated forms) of eEF1BδL and eEF1Bδ1 were abolished in the presence of PP1 (Figure 6A), and a similar result was obtained in the blots reacted with anti-phosphorylated eEF1Bδ antibody, illustrating that PP1 dephosphorylates both proteins at S499 and S133 *in vitro*, respectively. Together, this was confirmed using recombinant eEF1BδL, which was phosphorylated by CDK1, as the phosphorylated form of eEF1BδL was abolished by PP1 (Figure 6B).

We attempted to clarify the effects of eEF1BδL dephosphorylation on the interaction with its binding partner. As eEF1BδL interacts with Nrf2 to cooperatively activate the transcription of HSE-containing genes (Kaitsuka et al., 2011), we compared the levels of Nrf2 immunoprecipitated with eEF1BδL between WT and the S499 mutants. As previously illustrated, Nrf2 was detected in eEF1BδL WT-green fluorescent protein (GFP) and S499 mutant-GFP immunoprecipitates at the same levels (Supplementary Figure 2), illustrating that the dephosphorylation of eEF1BδL might not affect the interaction with its partners.

## Discussion

In this study, we revealed that eEF1BδL is dephosphorylated under heat shock and proteotoxic stress, and this dephosphorylated form has higher transcriptional activity. Its phosphorylation is mediated by CDK1, and its dephosphorylation is preferentially mediated by PP1.

The phosphorylation and dephosphorylation machinery is a fundamental system involved in cellular protein signaling and stress responses. Under heat shock stress, some kinases, and/or phosphatases are activated and/or inhibited, and they subsequently modulate the activity of heat-responsive proteins via phosphorylation and dephosphorylation, respectively. For example, some serine residues of heat shock factor 1 (HSF1) are phosphorylated during heat shock, and phosphorylation at S30 by calcium/calmodulin-dependent kinase II promotes its activation (Holmberg et al., 2001; Anckar and Sistonen, 2011). Furthermore, in response to heat shock, PP1 dephosphorylates the splicing factor serine and arginine rich splicing factor 10 (SRSF10), converting its function to splicing repression (Shi and Manley, 2007). During heat shock, PP1 dissociates from its inhibitor, nuclear inhibitor of PP1, and it can directly bind to SRSF10 protein. Based on our findings, it is speculated that a similar mechanism might exist for eEF1BδL dephosphorylation.

Dephosphorylated eEF1BδL displayed higher transcriptional activity on *HSPA6* HSE-containing reporters. However, the mechanism by which dephosphorylation affects this activity was not clarified in the current study. Sometimes, protein phosphorylation/dephosphorylation facilitates or inhibits its nuclear localization, degradation, DNA binding, and interaction with partner proteins such as HSF1 (Gomez-Pastor et al., 2018). Further studies are needed to clarify these findings. As another mechanism of eEF1BδL modification, RNF20/40 monoubiquitylates this protein at lysine 381 (In et al., 2019). Monoubiquitylation of eEF1BδL increases its accumulation and potentiates the recruitment of the transcription elongation factor p-TEFb to the promoter regions of HSE-containing genes.

We also demonstrated that canonical eEF1Bδ1 is dephosphorylated during heat shock stress. Phosphorylation of this protein at S133 by CDK1 is believed to decrease the translation elongation rate because its phosphorylation reduces its affinity for eEF1A (Sivan et al., 2011). Conversely, Monnier et al. (2001) revealed using a cell-free assay that the addition of CDK1/cyclin B to rabbit reticulocyte lysate decreased the elongation rate for valine, whereas the serine and phenylalanine elongation rate was increased in correlation with the phosphorylation of eEF1Bδ1. Therefore, the properties of S133-phosphorylated eEF1Bδ1 have not been fully unveiled. eEF1Bδ1 is also phosphorylated at T147 and S162 (Le Sourd et al., 2006). Phosphorylation at T147 is also mediated by CDK1; however, there is no report concerning the effect of this modification on translation elongation. Phosphorylation at S162 is mediated by casein kinase 2; however, the functional role of its phosphorylation is unclear.

The function of other eEF1s is revealed to be regulated via phosphorylation by a number of serine/threonine protein kinase. Major kinase among them is protein kinase C (PKC), which phosphorylates eEF1A, eEF1Bα, and eEF1Bδ causing a two fold increase in the GDP/DTP exchange activity (Peters et al., 1995; Sasikumar et al., 2012). eEF1A is also reported to be phosphorylated by type I transforming growth factor β receptor (TβR-I) at Ser300 (Lin et al., 2010). This site is located near the region of interaction with aa-tRNA in eEF1A, therefore, its phosphorylation could disrupt those interaction and lead to a reduction in translation. Another unique post translational modification is lysine methylation of eEF1A. Several works demonstrated that mammalian eEF1A was found to be methylated on five lysine residues (Jakobsson et al., 2018). Interestingly, there are eEF1A-specific lysine methyltransferases (KMTs), and these KMTs mono-, di-, or tri-methylates lysine residues. However, the linking of such methylation to the process of mRNA translation have not been elucidated (Jakobsson et al., 2018). Recent studies revealed that eEF1A lysine methylation affects the ability of eEF1A to interact with specific aminoacyl-tRNAs and mediates their interaction with the translating ribosome, thus modulates protein synthesis with the translation of specific genes (Jakobsson et al., 2017, 2018; Malecki et al., 2017).

About the regulation of eEF1s by dephosphorylation, there is a few report on eEF1A. PP1 is associated with several regulatory proteins as myosin phosphatase targeting subunit 1 (MYPT1), Spinophilin and others which determine the specificity of PP1 because PP1 lacks inherent substrate specificity (Peti et al., 2012; Leslie and Nairn, 2019). One of these regulatory proteins, TGF-β inhibited membrane associated protein (TIMAP) is a PP1 regulatory subunit and reported to dephosphorylate eEF1A1 (Boratkó et al., 2015). TIMAP forms a complex with the catalytic subunit of PP1 (Csortos et al., 2008; Shopik et al., 2013) and was found to co-localize with eEF1A1 in the plasma membrane. Basically, phosphorylated eEF1A1 by RhoK localizes to cytosol and functions as translation elongation, suggesting TIMAP regulates extra-ribosomal function of eEF1A1 in the plasma membrane (Boratkó et al., 2015; Boratkó and Csortos, 2017). However, it has not been determined which regulatory protein mediates the recruitment of PP1 to eEF1BδL and its dephosphorylation, and needed to be clarified in the future study.

eEF1BδL has been implicated in neurodevelopmental disorders, as *EEF1D* mutations were identified in severe intellectual disabilities by two independent groups (Reuter et al., 2017; Ugur Iseri et al., 2019). These mutations occur in an exon specific for eEF1BδL, suggesting that canonical eEF1Bδ1 is normally expressed in these patients. Meanwhile, eEF1Bδ1 has been implicated in several cancer types (Veremieva et al., 2014; Hassan et al., 2018). eEF1Bδ1 is overexpressed in osteosarcoma, and inhibition of this protein inhibits cell proliferation (Cheng et al., 2018). Therefore, our study indicated that the kinases and phosphatases of eEF1Bδ could be targeted to treat such diseases.

## Data Availability Statement

The original contributions presented in the study are included in the article/Supplementary Material, further inquiries can be directed to the corresponding author/s.

## Ethics Statement

The animal study was reviewed and approved by The Laboratory Animal Care and Use Committees of Kumamoto University.

## Author Contributions

TK and MM conceived this study and designed experiments. TK performed most of the experiments. KT provided critical advice. TK and MM wrote the paper. All authors contributed to the article and approved the submitted version.

## Conflict of Interest

The authors declare that the research was conducted in the absence of any commercial or financial relationships that could be construed as a potential conflict of interest.
