# Supplementary material for: Heat Shock-Induced Dephosphorylation of Eukaryotic Elongation Factor 1BδL by Protein Phosphatase 1
Source: Front Mol Biosci. 2021 Jan 14;7:598578. doi: 10.3389/fmolb.2020.598578 (PMC7841112; doi:10.3389/fmolb.2020.598578)
Supplement: Supplementary file 1 [file Data_Sheet_1.PDF]

Supplementary materials

Supplementary Figures

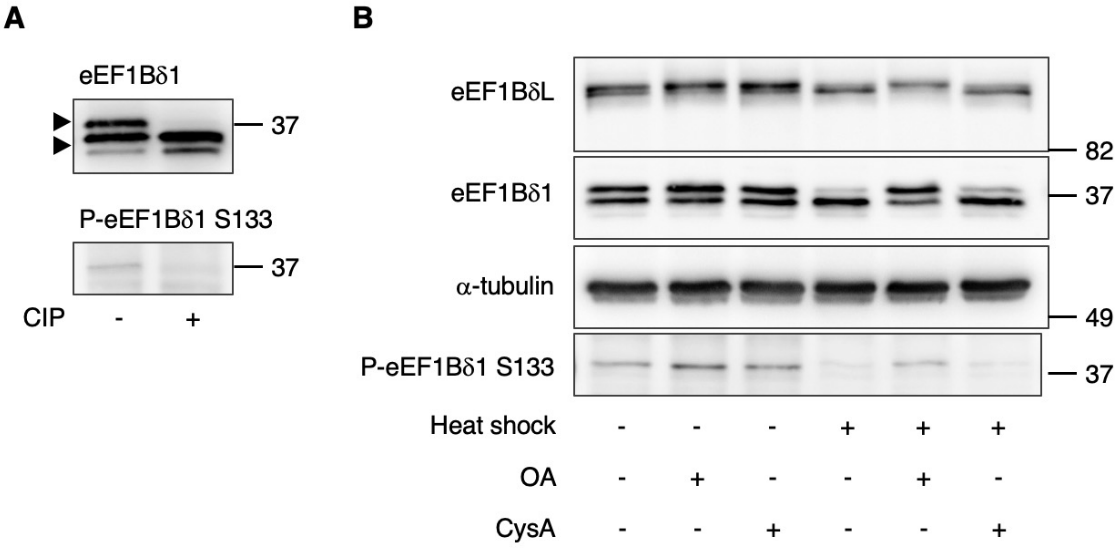

Figure S1. The anti-phosphorylated eEF1Bδ antibody react with upwardly shifted bands of eEF1Bδ1.

(A) Immunoprecipitated eEF1Bδ1 protein from cultured mouse neurons were treated with calf-intestinal alkaline phosphatase (CIP). The upper band disappeared following CIP treatment and was reacted with anti-phosphorylated eEF1Bδ antibody, indicated that the shifted band represents the phosphorylated eEF1Bδ1 at serine (S) 133. Similar results were obtained in three independent experiments.

(B) Cultured mouse hippocampal neurons were pretreated with 100 nM okadaic acid (OA) or 5 μM cyclosporine A (CysA) for 2 h followed by heat shock for 2 h. The upper band of eEF1Bδ1 was reacted with anti-phosphorylated eEF1Bδ antibody. Similar results were obtained in three independent experiments.

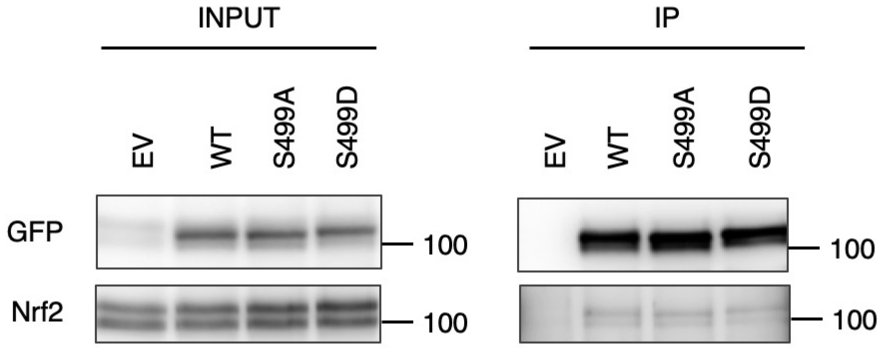

Figure S2. Co-immunoprecipitation of eEF1BδL S499 phospho-mutants and Nrf2. HEK293 cells were transfected with the expression plasmid carrying S499 phospho-mutants of eEF1BδL protein tagged with GFP and treated with 10 μM MG132 for 6 hr. Then cells were lyzed and then subjected to immunoprecipitation with anti-GFP antibody. The levels of co-immunoprecipitaed Nrf2 were similar between WT and phospho-mutants. Similar results were obtained in three independent experiments.
